# In Memoriam—Henry Noah Martin, M. D.

**Published:** 1890-01

**Authors:** Wm. W. Van Baun

**Affiliations:** Secretary


### IN MEMORIAM—HENRY NOAH MARTIN, M. D.

At the regular monthly meeting of the Homœopathic Medical Society of the County of Philadelphia, held November 14th, 1889, the following resolutions were unanimously adopted :

WHEREAS—Having been called upon by the will of Providence to part with our esteemed companion and brother colleague, Henry Noah Martin, M. D., we respectfully offer the following resolutions:

*Resolved*—That during his life we recognized in him the qualities that make the true physician, the respected counselor, and the safe teacher; that in his numerous contributions, provings, teachings, and precepts, we acknowledge the deep thinker and conscientious worker in his chosen profession, and that in his death we feel that Homœopathy has lost one of its most earnest advocates, and our Society one of its most illustrious members.

*Resolved*—That we extend to his bereaved family our sympathy for the removal of the husband and father, and to the community at large, for the loss of a gentle, kind, and beneficent friend.

*Resolved*—That a copy of these resolutions be sent to his family; that they be published in the *Hahnnemanian Monthly* and HOMŒOPATHIC PHYSICIAN, of Philadelphia, and that they be entered on the journal of this Society.

(Signed), A. R. THOMAS, M. D.,
GEO. W. SMITH, M. D.,
C. MOHR, M. D.,
*Committee.*

By order of the President,
WM. W. VAN BAUN, M. D.,
*Secretary.*

